# Economic and Clinical Outcomes Resulting From the Stage 4 Chronic Kidney Disease Case Management Quality Improvement Initiative

**DOI:** 10.1097/NCM.0000000000000253

**Published:** 2017-09-11

**Authors:** Beverly Everett, Liana D. Castel, Matthew McGinnis, Amy Beresky, Rudolph C. Cane, Tasha Cooper, Rajesh K. Davda, Donna Farmer, Stella M. John, Denise L. Sollars, John F. Rausch

**Affiliations:** **Beverly Everett, MD, FACP,** holds an MD degree from Tufts University School of Medicine, completed her Internal Medicine residency and Nephrology Fellowship at the University of North Carolina at Chapel Hill, practiced medicine for 15 years before joining Cigna, and after 18 years retired in 2015.; **Liana D. Castel, PhD,** is Editor-in-Chief of Patient Related Outcome Measures (Dove Medical Press, Ltd), Adjunct Professor at the University of Mount Olive Tillman School of Business, and medical writer at Cigna. She holds a PhD degree from the University of North Carolina at Chapel Hill. Her research interests include outcomes and statistics.; **Matthew McGinnis, BS,** has been with Cigna more than 13 years and leads the analytics team supporting Cigna's pharmacy benefits management business. He holds a US Patent for an End of Life Predictive Model, a Master of Science degree from Northwestern University, Illinois, and Certificate in Public Health from University of Florida.; **Amy Beresky, MS**, holds a Master of Science in Statistics degree from University of Massachusetts Amherst. She has 8 years' experience in health care data mining, analytics, and predictive modeling, supporting Cigna's clinical programs, is fluent in Spanish, and has served on the Board of Directors and Environmental Committee in the residential community.; **Rudolph C. Cane Jr., MD**, is Medical Director for Cigna Healthcare, Department of Clinical Performance and Quality. He holds board certification in Internal Medicine, completed Internal Medicine residency at Franklin Square Hospital Center, surgical internship at St. Agnes Medical Center, Baltimore, MD, and holds an MD degree from the University of Maryland School of Medicine.; **Tasha Cooper, RN**, is a registered nurse who has been employed by Cigna for more than 20 years, holding various roles in the areas of Clinical Program Development, Case Management, Utilization Management, Disease Management, and Quality Accreditation and Compliance. Her career also includes more than 8 years of hospital and home health nursing experience.; **Rajesh K. Davda, MD,** is the National Medical Director for network performance and quality improvement at Cigna Healthcare. Dr. Rajesh is board certified in internal medicine, nephrology and clinical informatics. Before joining Cigna in 2012, Raj was in private practice for 22 years in South Carolina and Texas.; **Donna Farmer, BSN, RN, CCM,** has a clinical background in managing catastrophic illness and life care planning. She has been employed at Cigna since 2001 where she has worked as a case manager (CM), CM supervisor, and clinical program consultant.; **Stella M. John, BSN, RN,** started at Cigna in 2005 as a catastrophic case manager and chronic condition kidney case manager. Currently, she holds the position of clinical coach with Cigna Workforce Development. Her 19 years' clinical experience includes surgical and medical ICU with a cardiac focus and intermediate care unit manager.; **Denise L. Sollars, BSN, RN, CCM**, began her career with Cigna in August 2001. She is a certified nurse case manager and was part of the Case Management team for 12 years. She is currently a clinical consultant for the Mid-America Market. Before joining Cigna, Denise had 15 years' hospital clinical experience.; **John F. Rausch Jr., MD, FACP, FNKF,** is board certified in Internal Medicine, has served as Board of Directors Chairman, National Kidney Foundation of Arizona, founding member and current Board of Directors member, Cardio Renal Society of America, and Medical Director at Cigna, Clinical Performance and Quality, Total Health and Network.

**Keywords:** case management, end-stage kidney disease, end-stage renal disease, kidney disease

## Abstract

**Purpose of Study:**

Chronic kidney disease (CKD) is a costly and burdensome public health concern. The goal of this study was to evaluate the impact on outcomes and utilization of a pilot program to identify and engage beneficiaries with CKD at risk for progression from Stage 4 to Stage 5.

**Primary Practice Settings:**

A quality improvement initiative was conducted to assess the impact of case management on costs and outcomes among 7,720 Cigna commercial medical beneficiaries with Stage 4 CKD enrolled in the United States between January 2012 and October 2012.

**Methodology and Sample:**

Claims data were analyzed to compare 3,861 beneficiaries randomized to receive condition-focused case management with 3,859 controls, with follow-up through July 2013. After using an algorithm to identify beneficiaries at highest risk of progression, a case management team implemented, among those assigned to the intervention, an evidence-based assessment tool, provided education and follow-up, engaged nephrologists and other providers, and conducted weekly rounds. Primary outcome measures were hospital admissions, emergency department visits, nephrologist visits, dialysis, arteriovenous (AV) fistula creation, and total medical costs. Analysis of variance techniques were used to test group differences.

**Results:**

As compared with controls, intervention beneficiaries were 12% more likely to have fistula creation (*p* = .004). Intervention beneficiaries were observed to have savings of $199 per member per month (PMPM), *F* = 23.05, *p* = .04. This difference equated to 6% lower total medical costs in the intervention group. Savings observed were derived half from improved in-network utilization and half from reduced hospital costs.

**Implications for Case Management Practice:**

- The observed 12% increased rate of creation of AV fistulas and $199 (6%) decrease in PMPM cost between the intervention and control groups corresponded to a savings of more than $18 million in 2015 U.S. dollars (USD).
- On the basis of observation of substantial improvements in outcomes and cost savings, health plan administrators could better serve those at highest risk of progression by implementing focused case management.
- Our findings support the value of care coordination between nephrologists, providers, and health plan case managers in improving outcomes and reducing total medical costs among beneficiaries at risk for CKD progression from Stage 4 to Stage 5.

Chronic kidney disease (CKD) is a costly and burdensome public health concern (Eknoyan et al., 2004), given the expense and morbidity associated with disease progression (Murphy et al., 2016). Overall, the prevalence of CKD (Stages 1–5) in the U.S. adult general population was 14.8% from 2011 to 2014 (United States Renal Data System, 2016), with the highest prevalence of Stage 3 CKD (United States Renal Data System, 2016). In 2014, patients with kidney disease in the United States had more than 4.6 million hospital outpatient or office-based provider visits, 1.1 million emergency department (ED) visits, and 3.1 million prescribed medicines (Agency for Healthcare Research and Quality, 2014). Medicare spending for beneficiaries aged 65 years and older with CKD rose from $14 billion in 2003 (Robbins, Kim, Zdon, Chan, & Jones, 2003) to exceed projections of $28.3 billion in 2010 (Xue, Ma, Louis, & Collins, 2001), and exceed $50 billion in 2014, representing 20% of all Medicare spending in this age group. CKD costs, although lower for an individual patient with CKD Stage 3 or 4, are higher in total due to a much higher prevalence of CKD Stages 3 and 4, and these patients have about an equal risk of progressing to end-stage renal disease (ESRD) or death due to cardiovascular events (Szczech et al., 2014).

Research is needed on the effectiveness of health plan initiatives to identify and manage patients at risk for progression in accordance with evidence-based guidelines (Kidney Disease: Improving Global Outcomes [KDIGO] CKD Work Group, 2013). Current clinical practice guidelines recommend that community-based CKD management programs include the following components:

- Disease monitoring;
- Integration with other chronic disease management programs including diabetes, hypertension, and heart failure;
- Medication management and dietary advice;
- Anemia management programs;
- Vaccination programs;
- Information and psychosocial support;
- Renal replacement therapy (dialysis and transplant) education; and
- Advanced care planning and end-of-life care where appropriate. (KDIGO CKD Work Group, 2013)

Benefit plans, insurers, and providers can play a key role in implementing clinical programs to incentivize more cost-effective care and in-network utilization, toward reducing morbidity, mortality, and costs.

The objective of this study was to evaluate the impact on outcomes and utilization of a pilot clinical management intervention conducted as part of a quality improvement initiative. Cigna undertook the initiative to identify and engage commercial customers with CKD at risk for progression from Stage 4 to Stage 5. Our goals were to identify those health plan beneficiaries at risk of imminent progression to Stage 5 within 6 months, to conduct new condition-focused case management outreach prior to the start of dialysis, and to assess the impact of this initiative on costs and outcomes (see Box 1).

**Table:** BOX 1 CKD Stages by GFR Category

Stage	GFR (ml/min/1.73 m^2^)	GFR Category
Stage 1	≥90	Normal or high
Stage 2	60—89	Mildly decreased^a^
Stage 3	30—59	Moderately decreased
Stage 4	15—29	Severely decreased
Stage 5	<15	Kidney failure

*Note*. In the absence of evidence of kidney damage, neither Stage 1 nor Stage 2 meets CKD criteria. CKD = chronic kidney disease; GFR = glomerular filtration rate. From “KDIGO 2012 Clinical Practice Guideline for the Evaluation and Management of Chronic Kidney Disease,” by Kidney Disease: Improving Global Outcomes (KDIGO) CKD Work Group, 2013, *Kidney International Supplements, 3*, pp. 1–150. Copyright 2012 by KDIGO. Adapted with permission.

^a^Relative to young adult level.

## Methods

### Study Design

As part of a quality improvement initiative incorporating existing evidence in support of care coordination (Berwick, Nolan, & Whittington, 2008; Chen et al., 2012), eligible health plan beneficiaries were randomized to receive condition-focused case management/care coordination services in order to assess the value of our identification, outreach, education, and management program with regard to outcomes, costs, and utilization. The study was determined exempt by Western Institutional Review Board in accordance with Office of Human Research Protections guidance on Health and Human Services regulations at 45 CFR 46.102(d).

### Study Population

Beneficiaries enrolled from January 2012 to October 2012 were identified as having CKD Stage 4. International Classification of Diseases (ICD)-9 code 585.4 was used to identify Stage 4, and ICD-9 codes 585.5 and 585.6 were used for Stage 5/ESRD. Identification as CKD Stage 4 required at least two estimated glomerular filtration rate laboratory results in the Stage 4 range (15–29) in the past year, with measurement dates more than 30 days apart and/or at least one diagnosis of CKD Stage 4 (on a medical claim) in the past year. In addition, individuals must not have had any indication (such as diagnosis) of having already progressed to CKD Stage 5/ESRD.

### Analysis

The risk predictive model score was based on known and identified progression risk factors (KDIGO CKD Work Group, 2013) including (in order of significance) age, presence/absence of prescription fill for calcium channel blocking agents, most recent calcium laboratory value (where available), presence/absence of a gap in care for nephrology consultation, presence/absence of episode treatment group (ETG) indicator for congestive heart failure, and most recent episode risk group (ERG) prospective risk score. OPTUMInsight's (formerly Ingenix) ERG methodology relies on patient underlying medical conditions, the source of which are the ETGs produced by the software (OPTUMInsight, 2012). Model weights were derived for the predictors identified in prepilot analysis of Cigna beneficiary data. During model development, all effects for the risk predictive model were significant at the .01 level. To be included, beneficiaries had to be a Cigna customer, eligible for benefits, at least 18 years of age, not have opted out of contact from Cigna communications, and have a risk predictive model score of more than 0.1.

**FIGURE 1:**
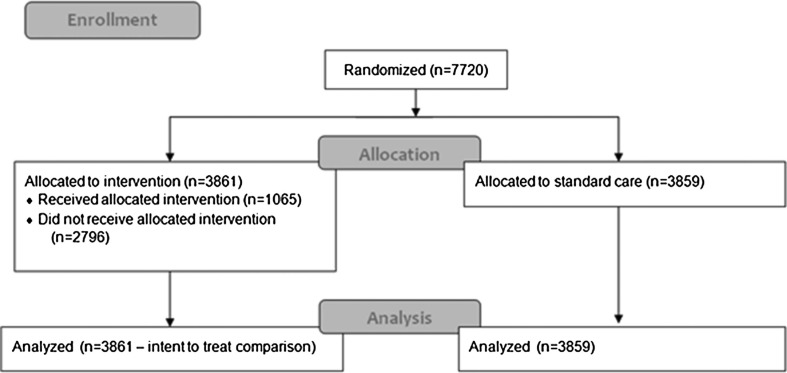
Flow diagram of study inclusion and exclusion criteria.

The PROC SURVEY SELECT procedure in SAS (SAS Institute Inc., Cary, NC) was used to randomize eligible beneficiaries from our identified cohort into either the control group (*n* = 3,859) or the intervention group (*n* = 3,861). See Figure 1. Analysis of variance techniques were used to test group differences.

### Study Intervention

The intervention consisted of the following components developed and implemented over 18 months: (a) an evidence-based assessment tool; (b) outreach protocols and scripts; (c) training of two dedicated staff case managers; and (d) weekly clinical rounds on CKD cases. Dedicated case managers engaged customers to proactively assess their plan of care, address gaps or risk factors, provide education on CKD, discuss Cigna benefits, review contracted places of service for dialysis care when needed, and provide other case management support as needed. Case managers completed (a) an assessment of medical history, the kidney disease stage and current plan of care, including diet, medications and adherence, functional deficits, pain assessment, sick-day plan, treatment plan, and care plan goals; (b) an assessment of risk factors and gaps in the plan of care; (c) an assessment of customer engagement with a nephrologist; (d) an assessment of employment and disability needs; (e) identification and management of comorbidities, such as hypertension and diabetes; and (f) an assessment of future case management needs. Case managers asked permission in their first call to the beneficiaries to speak about their medical issues; case managers documented the verbal approval before proceeding. Case managers conducted fax outreach and telephone calls to providers. The fax provided nephrologist offices with a customer-specific list of benefits and available programs, including information about in-network dialysis facilities close to the customer, and requested clinical information, including treatment plan, laboratory values, and medications. A detailed example, titled *CKD Management Case Study Vignette*, is given in the Appendix.

On the basis of the individual treatment plan, assessment, and findings, case managers provided the following:

- CKD education, nutrition, medication adherence, and over-the-counter supplements used impacting their condition, diagnostic testing importance, treatment modalities and options, and transplant potential;
- Evidence-based medical information and resources that assist in facilitating an informed decision-making process if the member must make a choice between dialysis and a transplant;
- Education on available coverage (e.g., benefits, co-pays, coverage for using in-network vs. out-of-network providers);
- Importance and reinforcement of physician's treatment plan and sick-day plan;
- Education and direction to contracted nephrologists if the member is not currently receiving services from a nephrologist; and
- Education and direction to contracted dialysis facilities, including the advantages of using an in-network facility.

Whenever possible, case managers engaged the member's treating physician, primary care physician, and/or nephrologist in the case management process. The clinical team also engaged the nephrologist and other health care professionals to coordinate care. Telephone assessments with beneficiaries lasted usually 60 min, and regular contact for educational sessions ranged from a single educational session to regular contact spanning over several weeks or months, in accordance with individual treatment plans. For particularly complex cases involving chronic comorbidities, conditions, and/or behavioral health issues requiring extensive case management beyond the scope of the intervention, beneficiaries were referred to other relevant programs.

Medical directors and medical case managers conducted weekly clinical rounds to discuss needed actions for individual customer needs. Accordingly, case managers completed referrals to pharmacy partners to address medication interactions, cost, and nonadherence, in collaboration with treating physicians. Case managers also addressed referrals with behavioral health partners and coaching partners for lifestyle management and chronic condition support, identified nutritional referrals by assessment, and encouraged customer intervention. Communication to treating providers included individualized customer benefits, in-network dialysis centers in the customer area, dietary benefit, chronic condition and coaching programs available, and kidney transplant benefit and network.

### Study Outcomes

Study outcomes included the number of all-cause inpatient admissions, all-cause ED visits, nephrologist office visits, dialysis visits, arteriovenous (AV) fistula creation, total claim cost in 2015 USD, and per member per month (PMPM) cost in 2015 USD. AV fistula creation is an outcome of interest because proactive AV fistula creation allows for maturation time of several weeks, ensuring access in time for dialysis. Preperiod metrics were calculated to ensure that the groups (control and treatment) were equivalent after randomization, whereas postperiod metrics were used to measure the impact of the CKD interventions. Final results were calculated using a difference-in-difference methodology to account for any slight preperiod differences between the groups.

All events were captured via administrative claims paid under each beneficiary's Cigna coverage, and there was no exclusion of claim events; unrelated events were assumed to be balanced through randomization. Costs were capped at $200,000 per member per year to mitigate the influence of outlier event costs.

### Statistical Analysis

On the basis of PROC POWER calculations in SAS, an estimated total of 4,946 beneficiaries were needed, with a one-tailed α of .05 and a (1 −β) of .80, to compare two independent proportions, given an absolute decrease of 5% in PMPM costs. The analysis was intent-to-treat, and thus included all randomized beneficiaries, regardless of disposition. Baseline characteristics were reported by group using frequency distributions and descriptive statistics. Analyses were conducted using SAS (Version 9.2; SAS Institute Inc., Cary, NC).

## Results

### Study Population

A total of 11 million beneficiaries were screened for eligibility. A total of 7,720 met eligibility criteria and were randomized between January 2012 and October 2012 (see Figure 1). Among those allocated to receive the intervention (*n* = 3,861), 1,065 (28%) randomized beneficiaries received the case management intervention; 3,859 beneficiaries in the control arm received standard care. There were no differences between the intervention and control groups in baseline demographics and clinical characteristics (see Table 1).

**TABLE 1:** Beneficiary Characteristics Prior to the Pilot Period

	Intervention		Control	
Characteristic	*n*	Mean (*SD*) or %	*n*	Mean (*SD*) or %
Age	3,861	59.9 (15.5)	3,859	59.7 (15.9)
Female	1,728	45%	1,752	45%
ERG score (retrospective risk score)	3,861	11.8 (10.6)	3,859	11.8 (10.7)
Risk PM score	3,861	0.128 (0.101)	3,859	0.129 (0.097)
PMPM medical cost (preintervention)	3,861	$2,691 ($6,433)	3,859	$2,659 ($5,959)

*Note.* ERG = episode risk group; PM = predictive model; PMPM = per member per month.

### Findings

Outcomes assessed included number of admissions, number of ED visits, number of nephrologist visits, number of dialysis visits, AV fistula creation, total claim cost, and PMPM cost. None of the preperiod differences were statistically significant between the intervention and control groups. Compared with controls, intervention beneficiaries were 12% more likely to have AV fistula creation (*p* = .004). Intervention group beneficiaries were observed to have savings of $199 PMPM; this difference equated to 6% lower total medical costs in the intervention group (*F* = 23.05, *p* = .04; see Table 2). Savings observed were derived half from improved in-network utilization and half from reduced hospital costs.

**TABLE 2:** Pre-/Postintervention (January 2012–October 2012) Differences by Group

	Control	Intervention	Net Difference	*F* (ANOVA)	*p* (ANOVA)
Admissions/1,000	−34.0	−47.4	13.4 (2%)	1.33	.37
ED visits/1,000	−220.6	−187.2	−33.4 (−4%)	0.01	.92
Nephrologist visits/1,000	1,151.2	1,390.6	239.4 (4%)	0.01	.93
Dialysis visits/1,000	11,305.6	11,138.3	167.3 (1%)	0.00	.99
AV fistula	58	67	8 (12%)	242.48	.004*
PMPM cost	−$507	−$308	$199 (6%)	23.05	.04*

*Note*. ANOVA = analysis of variance; AV = arteriovenous; ED = emergency department; PMPM = per member per month.

*p < .05.

## Discussion

As noted, CKD is a very costly and burdensome health condition. The goal of this study was to improve outcomes and lower costs for commercial customers at risk for CKD progression. Following a focused case management quality improvement intervention, claims data analyses showed 12% increased creation of AV fistulas and $199 (6%) decrease in PMPM cost between the intervention and control groups (corresponding to a savings of more than $18 million in 2015 USD).

This pilot demonstrated feasibility of identifying customers with CKD Stage 4 at risk for progression, provided an idea of real-world participation rates, and demonstrated improvement in outcomes and costs even despite a 28% participation rate. Our implementation of condition-focused case management led to increased AV fistula creation and PMPM savings and was consequently rolled out to all Cigna commercial customers with case management benefits who met the risk criteria.

Prior studies regarding the impact on outcomes and costs of CKD case management have had mixed results (Barrett et al., 2011; Hopkins et al., 2011). Some key features of the Cigna program that may have made it successful were identification of high-risk beneficiaries most likely to benefit, educational outreach about benefits to both beneficiaries and providers, and focused telephonic means of contact. One strength of this study was randomization, which allowed balance for potential unmeasured confounding factors. Because this analysis was intent-to-treat, we note that only 28% (1,065/3,861) of beneficiaries randomized to receive the intervention ultimately received it; thus, the effects on improving outcomes and reducing costs are likely understated.

This claims-based investigation of the effectiveness of a quality improvement intervention had certain limitations. Although clinical characteristics in the claims data were used to predict progression, future analyses would be strengthened by inclusion of chart data to both further track progression of CKD and account for the presence and severity of comorbidities. Observation of outcomes and costs over a period longer than a 10-month follow-up could perhaps show differences in other outcomes such as hospitalizations and ED visits that were not significant in the present analysis.

The Stage 4 CKD case management intervention study was created and designed to proactively identify, outreach, and engage a medically complex and costly group of Cigna beneficiaries. Substantial improvements in outcomes and cost savings were observed, implying that individuals at highest risk of progression can be better served by focused case management.

## APPENDIX: Chronic Kidney Disease (CKD) Management Case Study Vignette

**Table:**

The case manager was assigned a 62-year-old man with CKD Stage 4. He had diabetes, hypertension, and cardiovascular disease. He had diabetes since he was 30 years old. He had a primary care physician, a cardiologist, and a nephrologist within the same medical group. He saw his nephrologist every 6 months. An informational fax was sent to the nephrologist with the customer's Cigna programs available, gaps in care identified, in-network dialysis centers in the customer's area, information regarding LifeSource Program, and the website to find in-network providers. The fax also requested the physician's current treatment plan, next office visit, medical history, and latest laboratory results to assist the case manager in supporting the MD's plan of care. The nurse case manager outreached to the customer and left a voice message to call her back. She also sent a mail contact letter to the customer with her phone number and pertinent Cigna information. The case manager attempted another call to the customer and was able to complete an assessment specific to CKD Best Practices. His blood glucose and hypertension were stable; he was taking his medication as ordered; and he was attending all his MD appointments. The case manager assessed his current diet, discussed the renal diet with him, and gave him information regarding Cigna benefits he was not aware of to assist with losing weight. The case manager also gave him information regarding his dietician benefits and how to start meeting with a dietician. The case manager discussed the nephrotoxins to avoid and the importance of following his MD's treatment plan and following his diet and exercise on a regular basis to assist in keeping his kidney function stable. The case manager addressed possible gaps in care for his disease management and preventive care. The case manager asked the customer if there was anything regarding his health he would like to improve or change. He told her he wanted to lose weight. He expressed interest in participating in the Cigna program available in his benefits for weight loss. The case manager also discussed his medications with him and sent a request to Coach RX for a medication consult. The Coach RX identified from a cost perspective he could save money by filling his medications via Home Delivery and using a free preferred brand blood glucose meter with lower cost test strips. The case manager was able to proactively educate the customer regarding in-network dialysis centers in his area and the Cigna LifeSource Program. The case manager was able to discuss applying for Medicare as a secondary insurance once dialysis started. He was currently working full-time, so the case manager did not discuss return-to-work issues.
Follow-up call at a later date: The case manager sent in a referral to Cigna's Healthy Steps to Weight Loss program; the customer was currently participating and had lost 10 lb. He had a recommended diet from his MD and had met with a dietician twice. He signed up with Cigna Pharmacy and was saving money monthly on his medication costs. The customer did not need dialysis or transplant services at that time but was better ready and educated to face this hurdle if or when the time arises.
